# Understanding the Interplay Between Pore Structure and Ionic Liquid Interaction on the Gas Uptake of Microporous Carbons

**DOI:** 10.1002/smll.202501928

**Published:** 2025-08-11

**Authors:** Merve Ayyildiz, Kai Hetze, Konstantin Schutjajew, Purushottam Poudel, Renzo M. Paulus, Felix H. Schacher, Jan Dellith, Ulrich S. Schubert, Martin Oschatz

**Affiliations:** ^1^ Institute for Technical and Environmental Chemistry (ITUC) Friedrich‐Schiller‐University Jena Philosophenweg 7a 07743 Jena Germany; ^2^ HIPOLE Jena (Helmholtz Institute for Polymers in Energy Applications Jena) Lessingstrasse 12‐14 07743 Jena Germany; ^3^ Helmholtz Zentrum Berlin (HZB) Hahn‐Meitner‐Platz 1 14109 Berlin Germany; ^4^ Laboratory of Organic and Macromolecular Chemistry (IOMC) Friedrich Schiller University Jena Humboldtstraße 10 07743 Jena Germany; ^5^ Center for Energy and Environmental Chemistry Jena (CEEC Jena) Friedrich‐Schiller‐University Jena Philosophenweg 7a 07743 Jena Germany; ^6^ Department Competence Center for Micro‐ and Nanotechnologies Leibniz Institute of Photonic Technology Albert‐Einstein‐Straße 9 07745 Jena Germany

**Keywords:** activated carbons, confinement, ionic liquids, ionic liquid‐carbon interaction, nitrogen sorption

## Abstract

Interfaces formed between porous carbon materials and ionic liquids (ILs) play an essential role in catalysis as well as in electrochemical energy storage and energy conversion. Profound knowledge about the formed local structures on a molecular level is essential to create the desired physicochemical environments and to control the (remaining) porosity of the involved carbon materials. In the present study, the interplay between pore structure and ionic liquid (IL) loading in CO_2_‐activated microporous carbons is investigated with a special focus on the gas adsorption properties of the remaining hybrid materials. Two activated carbon materials with distinct micropore sizes (AC30 and AC120) are loaded with two hydrophobic ILs, namely 1‐ethyl‐3‐methylimidazolium‐bis(trifluormethylsulfonyl)imid (EMIM TFSI) and tributyloctyl phosphonium‐tris(pentafluoroethyl) trifluorophosphate (P_4448_ eFAP), at varying contents. The results reveal that IL configuration within the pores is crucial for gas uptake: EMIM TFSI maintains open porosity in larger micropores (AC120) but completely fills the smaller ones (AC30), whereas P_4448_ eFAP forms closed porosity by covering pore openings, enhancing gas uptake in residual pores (e.g., for CO_2_ at 273 K). N_2_ sorption at 298 K highlights the pronounced confinement effect of EMIM TFSI in smaller pores, leading to significant gas uptake. X‐ray diffraction (XRD), small‐angle X‐ray scattering (SAXS), and differential scanning calorimetry (DSC) analyses confirm these configurations and show that high IL loading induces bulk‐like behavior. These findings demonstrate how ionic liquids can be used to steplessly modify pore structures and influence solid‐liquid‐gas interfaces, providing insights into tailoring properties such as gas uptakes, hydrophobicity, and other physicochemical characteristics by their interaction with porous carbon materials.

## Graphical abstract

The interplay between pore structure and ionic liquid (IL) loading in CO_2_‐activated microporous carbons is explored to examine their gas adsorption and interface properties. Two hydrophobic ILs with distinct sizes demonstrate unique configurations within different pores, resulting in enhanced gas uptake and confinement effects. These findings provide valuable insights for tailoring hybrid materials for catalytic applications such as NRR and ORR.

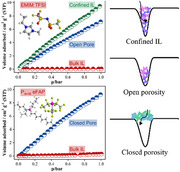

## 1 Introduction

Room temperature ionic liquids (ILs) and their local structures formed at the interfaces with solid materials are the subject of intense research.^[^ ^1^ ^]^ In particular, in the field of electrochemical energy storage and conversion, the combination of ILs and porous solids offers attractive options.^[^ ^2^ ^]^ For instance, interfaces between ILs and conductive high surface area electrode materials are applied for capacitive electrochemical energy storage, benefitting from the high voltage stability of ILs as electrolytes (e.g., in supercapacitors with high energy density).^[^ ^3^ ^]^ In the field of electrochemical energy conversion, that is, in electrically‐driven reactions, ILs are frequently applied as electrolytes as well due to their combination of ionic conductivity, high electrochemical stability window, and, often even more importantly, because of their outstanding properties as solvents.^[^ ^4^ ^]^

Confining ionic liquids in nanometric spaces, where dimensions are comparable to the size of their ions, alters their properties significantly when compared to the bulk phase. This allows the production of ILs with tailored physicochemical properties, supporting a large number of applications and attracting significant attention from many researchers.^[^ ^5^ ^]^ Weingarth et al.^[^ ^6^ ^]^ studied the thermal behavior of ionic liquids confined within the nanopores of a carbon material for supercapacitor applications. Using differential scanning calorimetry (DSC) measurements, they demonstrated that the freezing and melting points of the confined ionic liquids are significantly altered compared to their bulk state. Ratajczak et al.^[^ ^7^ ^]^ also investigated the thermal behavior and mobility of 1‐ethyl‐3‐methylimidazolium bis(fluorosulfonyl)imide (EMIM FSI) confined within micro‐ and mesoporous carbons using DSC and nuclear magnetic resonance (NMR) techniques. They found that when ionic liquids are confined in microporous carbon, the strong interaction between the IL and the pore walls results in reduced ion mobility. The pore size was additionally found to have an impact on the ILs ion dynamics. High gas adsorption capacity is a well‐known feature of ionic liquids, which can be further enhanced through confinement effects. Harmanli et al.^[^ ^8^ ^]^ investigated the adsorption properties of confined ionic liquids, specifically examining N_2_ gas uptake when 1‐ethyl‐3‐methylimidazolium acetate (EmIm OAc) is confined within micro‐ and mesoporous carbons, both with and without nitrogen doping, demonstrating enhanced N_2_ uptake. A related study by Uzun et al.^[^ ^9^ ^]^ showed that coating activated carbon with the ionic liquid 1‐butyl‐3‐methylimidazolium acetate (BMIM OAc) significantly enhanced CO_2_ selectivity over N_2_ and CH_4_ by altering surface polarity and creating new physisorption and chemisorption sites through nanoconfinement. Building upon these insights it becomes evident that not only the carbon has an impact on the IL's physicochemical properties but also in reverse conclusion the ionic liquid can be used as a means of modifying the carbon's surface functionality, porosity, and hence its reactivity and catalytic activity toward different reagents, which in turn can be utilized as catalyst systems for a series of applications.

One obvious example here is the field of the electrochemically‐driven reduction of nitrogen to ammonia (often denoted as nitrogen reduction reaction, NRR), which is currently intensely investigated due to its potential contribution to carbon‐free fertilizer production and the application of ammonia as hydrogen storage and carrier molecule.^[^ ^10^ ^]^ NRR is based on the reduction of dinitrogen with protons and electrons instead of elemental hydrogen coupled with an oxidation reaction on the counter electrode of an electrochemical cell.^[^ ^10^, ^11^ ^]^ This offers possible decentralized production schemes for ammonia without the need for carbon‐intense concepts for hydrogen production from natural gas and with energy input based on electricity from renewable sources. However, in addition to the chemical inertness of the strong N≡N bond, the low solubility of dinitrogen in the most‐often applied aqueous electrolytes and the orders of magnitude higher concentration of protons near the electrode surface are a serious limitation for the practical relevance of NRR.^[^ ^11^, ^12^ ^]^ The concurrent hydrogen evolution reaction (HER) is getting predominant, and the observed faradaic efficiencies (FEs) for ammonia production as well as the corresponding time‐related yields remain very low.^[^ ^10^, ^12^, ^13^ ^]^ At least if typical laboratory electrochemical cells such as H‐cells are applied, this problem of trace ammonia generation leads to significant deviations in results obtained between different laboratories and complicates analytical product quantification.^[^ ^14^ ^]^ This is the major reason why it remains difficult to identify ideal structural features within materials which make them qualified as catalysts for NRR. In addition to the use of alternative electrode and reactor concepts,^[^ ^15^ ^]^ application of IL electrolytes, specifically fluorinated ones with high nitrogen solubility, has been reported as an attractive option to overcome this challenge.^[^ ^16^ ^]^ For instance, MacFarlane and co‐workers achieved NH_3_ production at as high as 60% FE in P_6,6,6,14_ eFAP ionic liquid at room temperature and ambient pressure.^[^ ^10c^ ^]^

A related line of research revealed that ILs can also be specifically used to modulate the catalytically active interface formed between electrodes and electrolytes. In the “supported ionic liquid phase” (SILP) concept, catalysts are dispersed in thin layers of ILs on surfaces of porous materials.^[^ ^17^ ^]^ In the related “solid catalysts with ionic liquid layer” (SCILL) approach, thin layers of ILs are immobilized on solid catalysts.^[^ ^18^ ^]^ Both concepts are generally applicable for classical thermal catalysis but also for alternative routes of energy input such as electrically‐ or light‐driven reactions.^[^ ^19^ ^]^ They have in common that they aim to modify the microenvironment of (electro)catalysts and have already proven to have significant impacts on the stability, activity, and selectivity of electrocatalytic processes.^[^ ^9^, ^20^ ^]^ For electrochemical processes involving gaseous substrates to be converted, the central requirement for the successful use of such concepts is most often to achieve precise control over the formed 3‐phase boundaries between solid electrode, liquid electrolyte, and gaseous reaction partners. For instance, Etzold and co‐workers demonstrated for the electrochemical CO_2_ reduction reaction (CO_2_RR) that the IL 1‐butyl‐3‐methylimidazolium bis(trifluormethylsulfonyl)imid (BMIm  NTf2) can be used as a chemical trapping agent if deposited on a copper foam catalyst, which selectively suppresses the formation of ethylene, ethanol and *n*‐propanol with small impact on the formation of other products.^[^ ^21^ ^]^ However, whilst SILP and SCILL have been widely employed for CO_2_RR, their possible potential for NRR remains to be explored. In order to make optimal use of such IL‐based concepts in electrocatalysis, the 3‐phase boundaries have to be understood on the molecular level as they are playing a crucial role for the micro‐ and macrokinetics as well as the electron‐transfer processes occurring in electrochemical reactions.

From the perspective of electrode materials, porous carbons are often used as “support materials” to immobilize and to stabilize electrocatalytically active nanostructured metals.^[^ ^22^ ^]^ Another example is the emerging field of single metal‐site materials into which metal ions or atoms are stabilized into (in most cases heteroatom‐rich) porous carbon matrices. Such catalysts, thus, showcase the transition from homogeneous to heterogeneous catalysis and have shown remarkable catalytic properties in various reactions.^[^ ^10^, ^23^ ^]^ It is therefore not surprising that these materials are often combined with the use of ionic liquids either as electrolytes or by utilization of the SILP and SCILL concepts in electrocatalysis. These different carbon‐based electrode compounds can appear in various different porosities which, in turn, will significantly impact their interaction with ionic liquids as well as with surrounding gas molecules. In particular, when the pore sizes in microporous materials approach the molecular dimensions of the ionic liquids, special understanding about the adsorption states of ILs, possible changes of their physicochemical properties as well as the changes on the remaining pore structures or pore blocking must be developed to tailor such 3‐phase boundaries for a specific electrocatalytic application. Confinement of ILs or surface layer formation on carbon is associated with changes of the physicochemical properties of both compounds.^[^ ^5^, ^6^, ^7^, ^8^ ^]^ In addition, even if pores are apparently fully occupied with ions or if the pore entrances are blocked, free space can remain, which can lead to a remarkably high gas uptake and the possibility to control pore sizes and chemical environment of the adsorbate molecules.^[^ ^8^ ^]^ Both the formation of a liquid film around particles but also the surface binding of ions leaving empty pore space can be understood as what is otherwise referred to as “porous ionic liquids” or for other liquids as “porous liquids”.^[^ ^24^ ^]^ What distinguishes the present study from most of the previous works on porous (ionic)liquids is that we are focusing on keeping the materials which can be handled as solids. For instance, after the loading of ILs, they could be engineered into electrodes or applied as solid catalysts.

In this regard, a series of two activated carbon materials and their interaction with two different ionic liquids at various loadings is showcased in the present work. The configuration of the IL within the carbon support and the impact of the structures formed at the carbon‐IL interfaces on the nitrogen gas sorption properties of the hybrid materials are investigated. The study provides insight into whether the combination of carbon materials with different micropore sizes and two different ionic liquid results in true confinement within the pores or creates a porous liquid‐like environment. It also offers valuable perspectives on the tailored carbon‐IL interface that could be beneficial, for instance, in NRR applications, such as enhancing local N_2_ availability at the catalyst surface, which may help suppress the HER. From a more general perspective, our work shows exemplarily for carbon materials in which narrow windows for IL dimensions and carbon pore sizes, the formed interfaces can change from pore filling to the formation of closed pores. This research is thus also of high relevance for the emerging field of porous liquids. These insights might lead to generalizable design strategies which may help overcome the aforementioned limitations and potentially improve catalytic activity.

## 2 Results and Discussion

**Figure 1:**
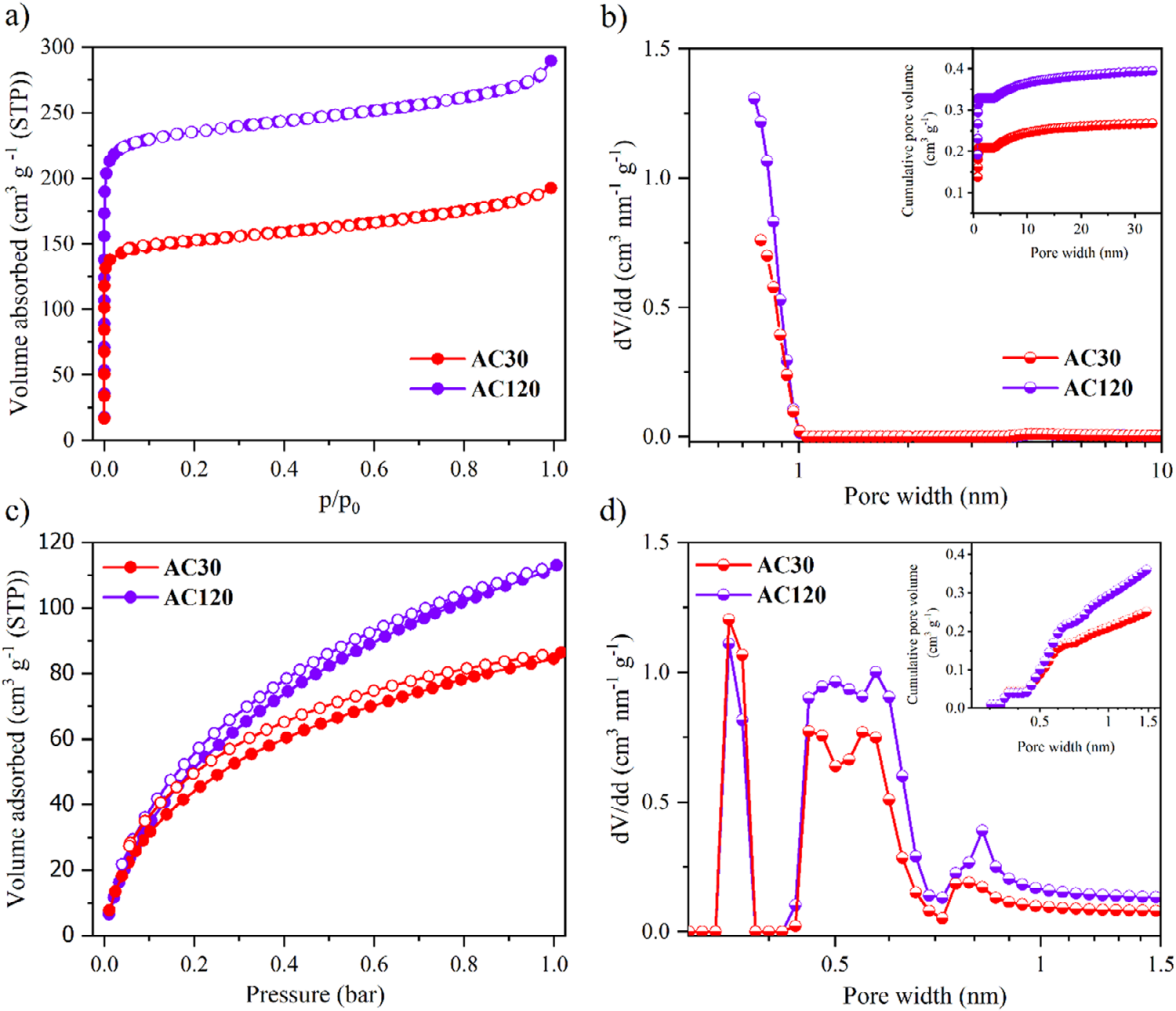
a) N_2_ physisorption isotherms measured at 77 K, adsorption and desorption branches are represented by filled (●) and open (○) circles, respectively, b) corresponding differential and cumulative pore size distributions of pure carbon materials, c) CO_2_ physisorption isotherms measured at 273 K, adsorption and desorption branches are represented by filled (●) and open (○) circles, respectively, d) corresponding differential and cumulative pore size distributions.

Two porous carbon materials with distinct micropore sizes are utilized in this study, namely AC120, which contains micropores of ≈0.8 nm, and AC30, with slightly smaller micropores with a diameter centered ≈0.7 nm (as determined from CO_2_ physisorption data). The porosity characteristics of these activated carbons are controllable by the duration of CO_2_ activation. **Figure** **1** presents the N_2_ and CO_2_ adsorption/desorption isotherms, along with the corresponding pore size distribution (PSD) profiles for each carbon sample. Both materials exhibit type I isotherms for N_2_ physisorption, indicative of a predominantly microporous structure, as shown by the sharp increase in adsorption uptake at low relative pressures. PSD analysis reveals that the majority of pores which are accessible via N_2_ physisorption has a diameter smaller than ≈0.75 nm. The total nitrogen accessible pore volume was determined to be 0.3 cm^3^ g^−1^ for AC30 and 0.4 cm^3^ g^−1^ for AC120 by using the Gurvich method.^[^ ^25^ ^]^ However, even though the difference in the inflection of the isotherms at low relative pressures indicates a difference in micropore size, due to the small diameter of the relevant pores it stands to reason that nitrogen sorption isotherms cannot sufficiently resolve the difference between the two carbons.

Given the smaller kinetic diameter of CO_2_ (3.3 Å) compared to N_2_ (3.6 Å), CO_2_ physisorption is particularly suited for analyzing ultramicropores, especially in the absence of heteroatoms on the sorbent's surface, as it is the case with these samples.^[^ ^26^ ^]^ The CO_2_ isotherms indicate that pores as small as 0.35 nm remain accessible. The effects of activation time are evident in both gas uptake and PSD data. Prolonged activation leads to increased adsorption capacity for both gases, while expanding the volume of larger pores, thereby contributing to a higher BET surface area (600 and 920 m^2^ g^−1^ for AC30 and AC120, respectively).

Considering the pore dimensions of the two samples are matching the ionic dimensions of typical ILs, the carbons provide a suitable environment for studying the expected specific effects of carbon/IL interactions on a molecular level. For this purpose, the interaction with two hydrophobic ionic liquids of different molecular sizes is examined. 1‐ethyl‐3‐methylimidazolium‐bis(trifluormethylsulfonyl)imid, (EMIM TFSI, smaller) and tributyloctyl phosphonium‐tris(pentafluoroethyl) trifluorophosphate, (P_4448_ eFAP, larger) are used at (nominal) loading ratios of 15%, 30%, and 50% based on the total pore volume of the carbon (Figure S1, Supporting Information). We have selected P_4448_ eFAP and EMIM TFSI for the reason of their high N_2_ solubility and to showcase in which narrow window of pore sizes in ionic liquid ion dimensions the formed interfaces can change in their properties as discussed in the following. Furthermore, it is known that imidazolium‐based ionic liquids exhibit high catalytic activity and gas solubility.^[^ ^27^ ^]^ Specifically, EMIM TFSI is a widely used ionic liquid in electroreduction reactions, such as the CO_2_ reduction reaction.^[^ ^28^ ^]^ Additionally, the relatively smaller ion size compared to P_4448_ eFAP makes it particularly suitable for comparing ILs of different sizes and for promoting local nitrogen enrichment in microporous environments relevant to this study.

**Figure 2:**
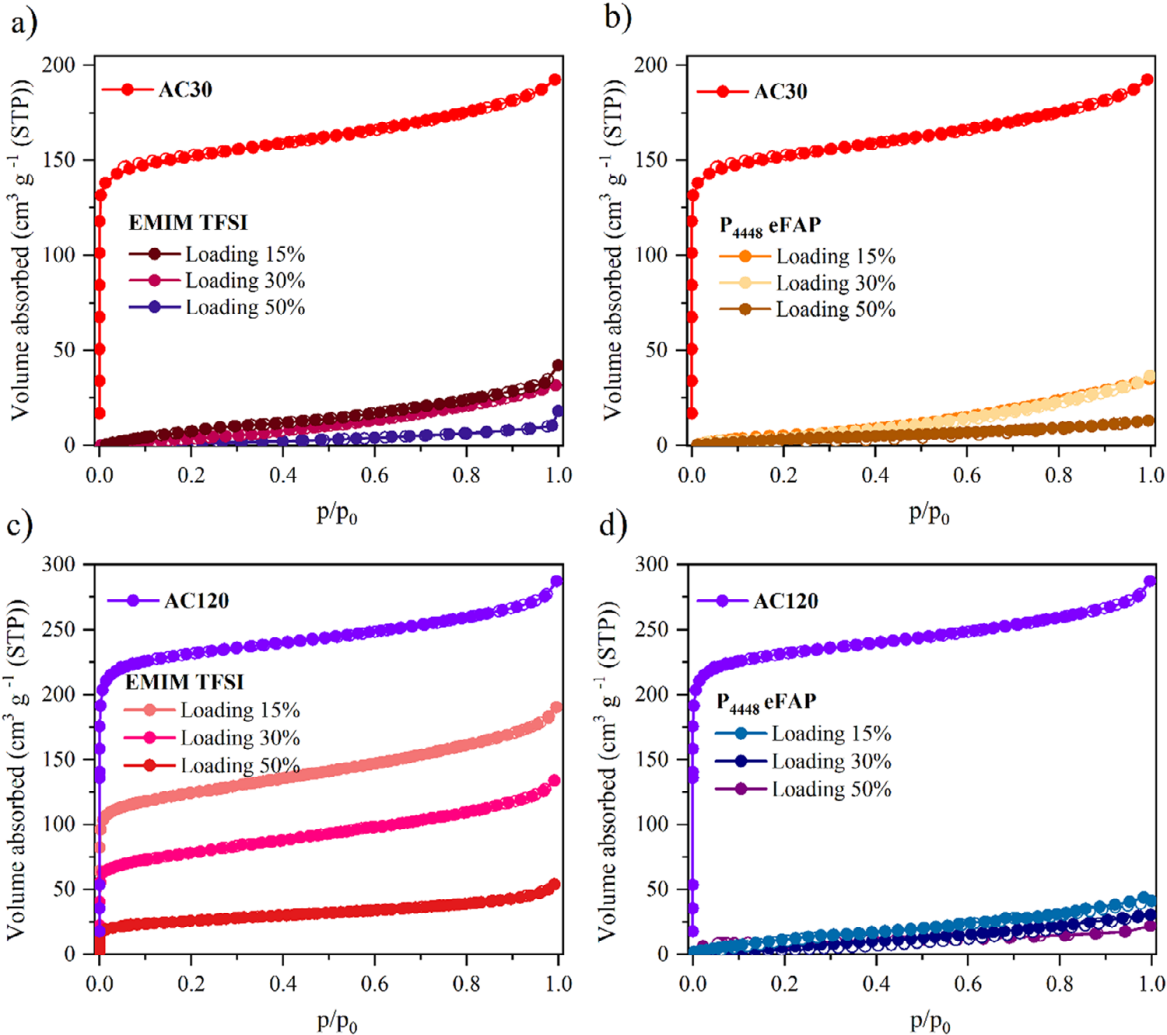
N_2_ physisorption isotherms measured at 77 K for hybrid materials adsorption and desorption branches are represented by filled (●) and open (○) circles, respectively. a) AC30 loaded with EMIM TFSI, b) AC30 loaded with P_4448_ eFAP, c) AC120 loaded with EMIM TFSI, and d) AC120 loaded with P_4448_ eFAP.

Examining the N_2_ physisorption of the carbon‐IL hybrid materials at 77 K is crucial for a comprehensive understanding of their porous characteristics and the interactions at the interface. ILs were introduced into AC30 and AC120 at the ratios described above. The N_2_ physisorption isotherms of the IL‐loaded materials are shown in **Figure** **2** and reveal that AC30 hybrid samples exhibit type II isotherms, with significantly reduced N_2_ uptake at any IL or loading level, suggesting a complete loss of open porosity for N_2_ at 77 K. AC120 hybrid samples display type II isotherms when loaded with P_4448_ eFAP, indicating a similar lack of open porosity under these conditions. However, when loaded with EMIM TFSI, type I isotherms persist in case of AC120, suggesting that this IL coats the pore surfaces or at least only partly occupies the pore volume while still maintaining accessible porosity within the material. The N_2_ uptake in these hybrid materials decreases as the loading level increases, which is expected as more IL occupies the pores. Notably, the extent of the decrease of nitrogen uptake is always higher than the loading degree, which was calculated based on the total pore volume, indicating an accommodation of IL‐ions leaving encapsulated voids or in other words, closed porosity. These results underscore a relationship between the pore size of the carbon and the size of the ionic liquid, highlighting the importance of optimizing pore‐filling percentages to maintain open porosity.

To assess the reproducibility of the gas adsorption measurements, selected samples (AC30, AC30–EMIM TFSI 30%, and AC30–P_4448_ eFAP 30%) were measured three times under identical conditions. Repeat measurements were conducted for N_2_ adsorption at 77 K, N_2_ adsorption at 298 K, and CO_2_ adsorption at 273 K. The resulting isotherms showed excellent agreement and are provided in the (Figure S2, Supporting Information).

**Figure 3:**
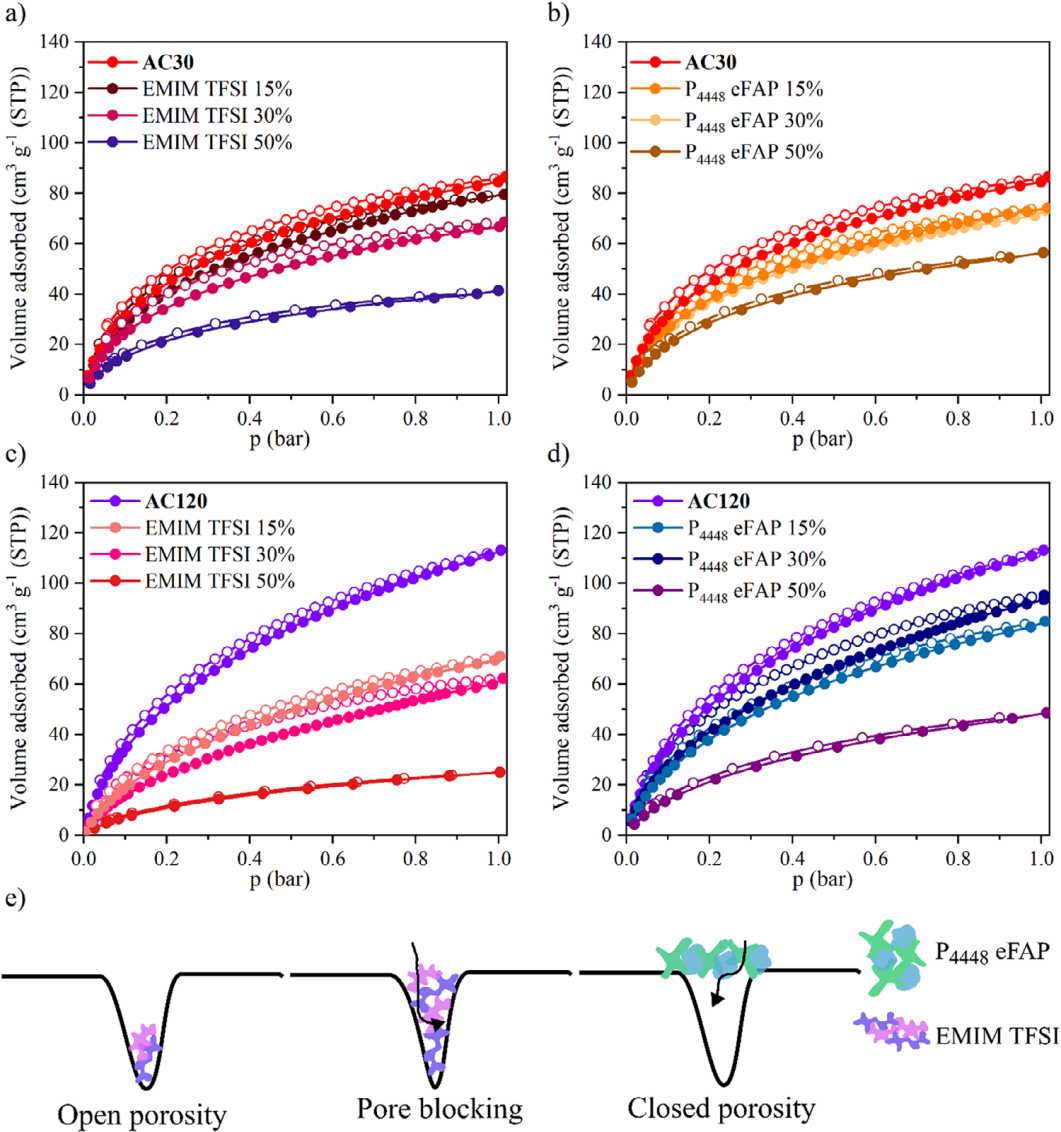
CO_2_ physisorption isotherms measured at 273 K, adsorption and desorption branches are represented by filled (●) and open (○) circles, respectively, for AC30 samples with a) EMIM TFSI loaded, b) P_4448_ eFAP loaded, and for AC120 samples with c) EMIM TFSI loaded, d) P_4448_ eFAP loaded, and e) schematic of ionic liquid positions in the pores; pore blocking structure for EMIM TFSI, closed porosity for P_4448_ eFAP, and open porosity for EMIM TFSI samples.

CO_2_ physisorption isotherms at 273 K are shown in **Figure** **3**, highlighting a clear porosity‐dependent trend in gas uptake. Samples with P_4448_ eFAP consistently exhibit higher gas uptake than those with EMIM TFSI. The highest uptake is observed for the AC120 sample, reaching up to 100 cm^3^ g^−1^. The amount of adsorbed CO_2_ decreases as the IL loading volume increases. In the case of AC30, both ILs seem to close off the pore openings and not to enter the pore space itself to any relevant extent. That is in accordance with nitrogen isotherms at 77 K, which resemble those of unporous solids. With CO_2_ isotherms at 273 K gas uptakes are directly linked to pore volume displaced by IL introduced as the adsorbate diffusivity and mobility if the IL ions are still sufficiently high to allow for occupation of free pore volume by the adsorbate. EMIM TFSI seems to be located in the internal pores of AC120, giving rise to similar and loading‐proportional uptakes and pore volumes in both, CO_2_ and N_2_ isotherms, whereas P_4448_ eFAP again accumulates at the pore entrances, yielding high CO_2_ uptakes at room temperature and type II nitrogen isotherms at 77 K. These observations provide insight into the interactions of ILs with the carbon surface, where each IL exhibits distinct behavior within different pore structures, adopting unique configurations: At low loadings (e.g., 15%), EMIM TFSI forms a thin layer covering the pore openings of AC30. As the IL loading increases (30% and 50%), capillary forces and the tendency of the IL to minimize surface tension cause the IL to be drawn into the pores, transitioning from surface coverage to complete pore filling. In contrast, P_4448_ eFAP closes off pore openings in both AC30 and AC120, forming closed porosity. For AC120, EMIM TFSI partly fills the pores remaining the open porosity (Figure 3e). The CO_2_ isotherms indicate that CO_2_ at the measurement temperature can fill the free volume between individual ions of the ionic liquid when confined within the pores, or, in the case of closed porosity, the gas can diffuse through the IL layer and fill the free volume within the ionic liquid itself.

For further investigation, scanning electron microscopy (SEM) and energy dispersive X‐ray (EDX) spectroscopy mapping were applied to examine the particle morphology, topology, and successful incorporation of ILs into the samples (Figures S3 and S4, Supporting Information). Slight indications of a smoother surface in IL‐impregnated samples can be recognized, the most important finding from these measurements, however, is that there are no evident free agglomerates of IL or contrary uncoated carbon particles, indicating the homogeneity of IL distribution across the carbon substrates.

**Figure 4:**
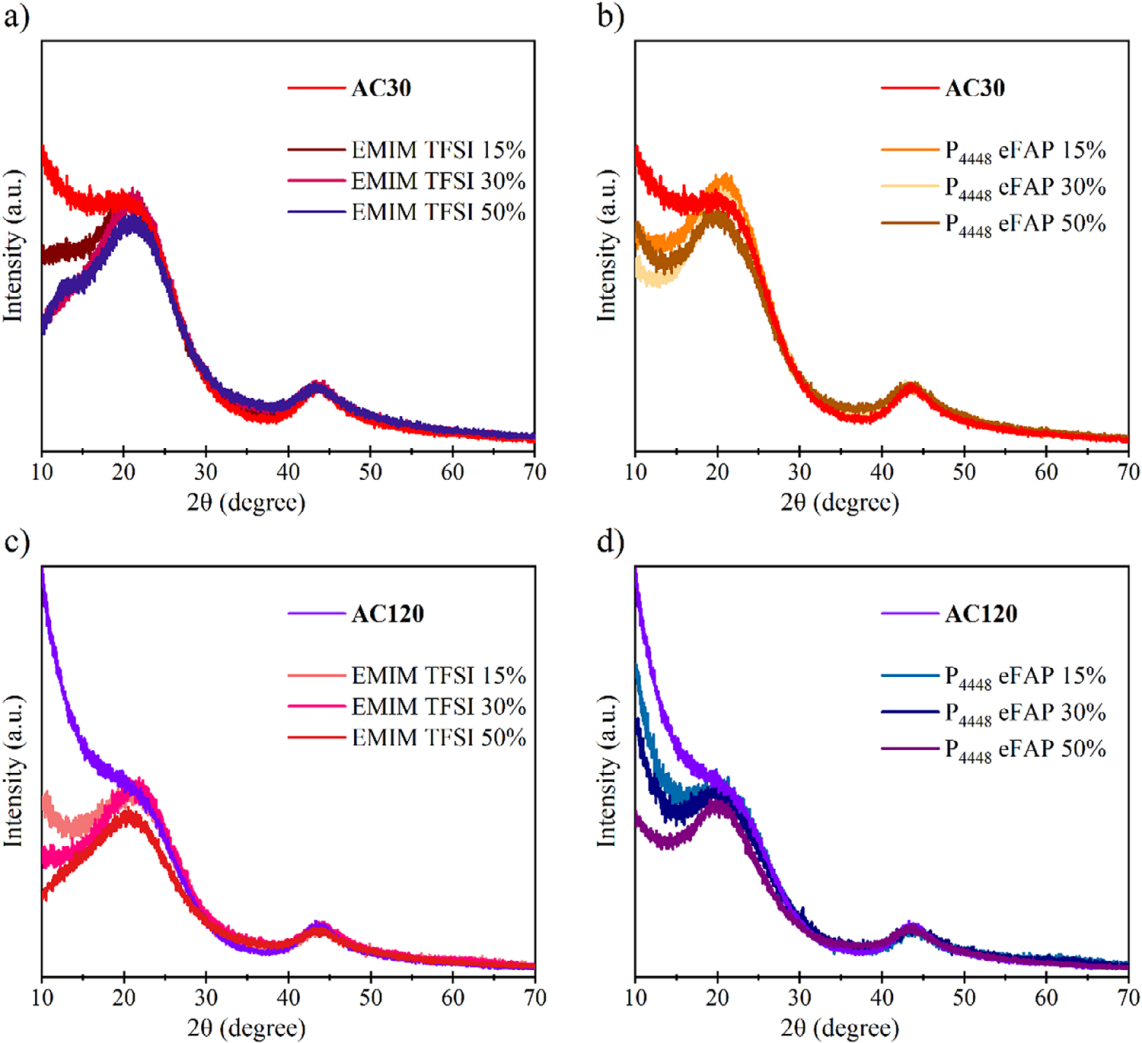
X‐ray diffraction patterns of a) AC30 loading with EMIM TFSI, b) AC30 loading with P_4448_ eFAP, c) AC120 loading with EMIM TFSI, d) AC120 loading with P_4448_ eFAP.

The structural characteristics of pure carbon and the hybrid materials were analyzed through X‐ray diffraction (XRD), as shown in **Figure** **4**. Pure activated carbons display broad diffraction signals located ≈2θ = 20° and 45°, corresponding to the (002) and (100) Bragg reflections, respectively. The broad shape and the absence of a well‐defined reflection at 20° suggest significant disorder in the stacking of graphene layers, indicating a predominantly amorphous structure as it is typical for carbons with a high micropore content. The low intensity reflection near 45° points to some in‐plane ordering of carbon atoms within graphene sheets, where the presence of graphitic domains is inferred.^[^ ^29^ ^]^ However, the broadness of the peak underscores an overall amorphous and disordered structure. Additionally, the high porosity of these carbons contributes to diffuse scattering at low angles, leading to a raised baseline ≈10°, which is particularly pronounced for AC120. The diffuse scattering can be used as a qualitative measure of remaining porosity, regardless whether it is gas accessible or not.

For the hybrid materials with P_4448_ eFAP, diffuse scattering is still present, associated with the left porosity from its closed pore structure, though this effect diminishes with increasing IL loading. Conversely, for AC30 with EMIM TFSI, the diffuse scattering is not observable for high loadings (30% and 50%), as no porosity remains due to its pore blocking structure, and a shoulder peak ≈12° emerges with higher EMIM TFSI loading. From the literature, it is known that for the liquid phase imidazolium based ionic liquids, some reflexes are observed in the X‐ray scattering. One broad main peak is located at 2θ =∼21.2° which is attributed to the both intramolecular interactions and close‐contact intermolecular interactions and a shoulder peak is at 2θ =∼12.7° which originates from crystal planes like [3,0,0] and [1,1, −1], representing shorter characteristic separations between polar groups not separated by long alkyl tails.^[^ ^30^ ^]^ From the XRD measurements of the ionic liquids (Figure S5, Supporting Information), it can be concluded that this peak appears at higher IL loadings, where the excess ionic liquid extends beyond the pores and spreads onto the external carbon surface. This effect is also observed in AC120, where the increased EMIM TFSI loading indicates IL occupation along the external surfaces of the carbon pores. These XRD results align well with the CO_2_ physisorption data, providing consistent evidence regarding IL configuration and pore accessibility in the materials.

**Figure 5:**
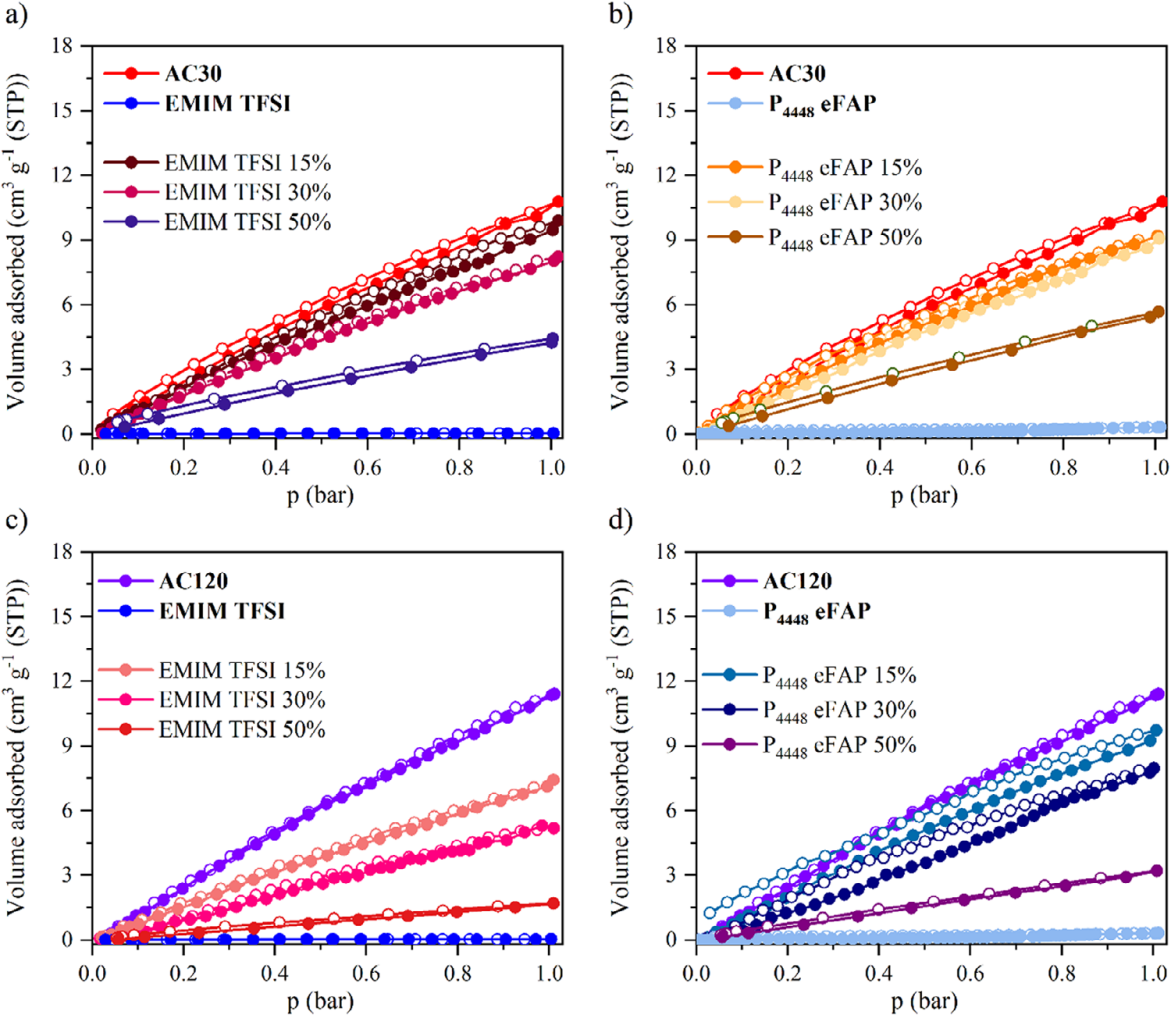
N_2_ physisorption at 298 K, adsorption and desorption branches are represented by filled (●) and open (○) circles, respectively, for ionic liquids and for AC30 a) loaded with EMIM TFSI, b) loaded with P_4448_ eFAP, as well as for AC120 c) loaded with EMIM TFSI and d) loaded with P_4448_ eFAP.

The nitrogen sorption isotherms at 298 K of the samples are presented in **Figure** **5**. The pure AC30 sample exhibits an uptake of ≈10.5 cm^3^ g^−1^, while the pure AC120 sample shows a slightly higher uptake of 11.5 cm^3^ g^−1^, attributed to its higher porosity. For all hybrid materials, as discussed above, gas can occupy the free volume within the ionic liquid, leading to high gas uptake. This behavior follows the trend observed in the CO_2_ sorption isotherms. Hybrid materials containing P_4448_ eFAP consistently exhibit higher gas uptake than those with EMIM TFSI, owing to the residual porosity from the closed pore structure, which allows more gas molecules to be accommodated. Additionally, high IL loadings result in decreased gas uptake, stepwisely approaching a behavior similar to that of bulk IL. These results are in good agreement with the XRD analysis, which also demonstrates that high IL loadings lead to bulk IL behavior in the samples. Preliminary findings from our yet unpublished research show that the confinement of ions within smaller pores enhances N_2_ uptake at room temperature. This is also evident in Figure 5, where AC30 hybrid samples exhibit higher uptake compared to the AC120 hybrids.

**Figure 6:**
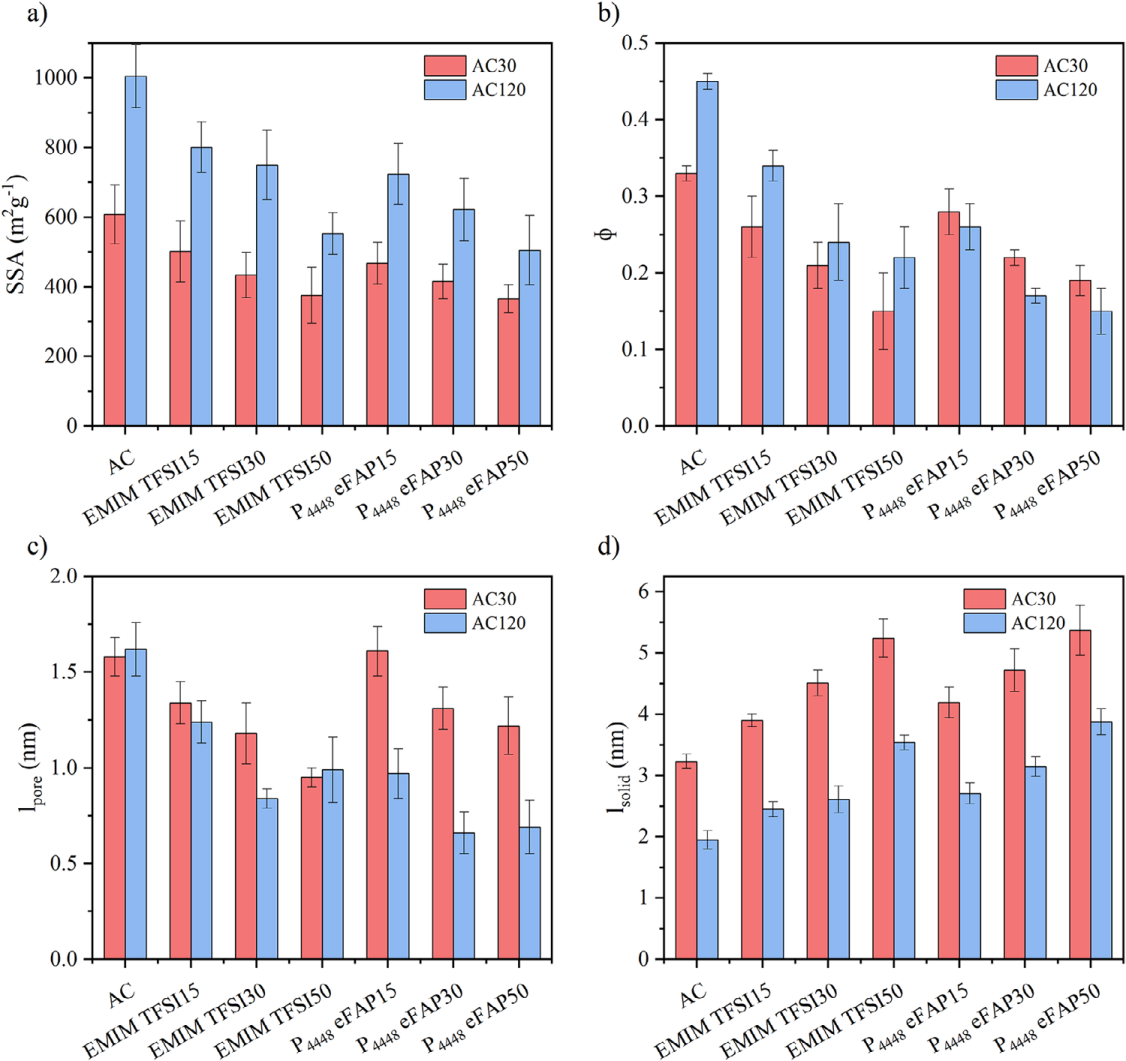
Parameters obtained from SAXS measurements for pure carbon and the hybrid materials: a) surface area, b) porosity, c) average chord length of the pores (*l*_pore_), and d) average chord length of the walls (*l*_solid_).

The hybrid materials were further analysed using small angle X‐ray scattering (SAXS, Figures S6–S8, Table S1, Supporting Information). Detailed calculations from SAXS measurements can be found in the Supporting Information. **Figure** **6** presents parameters obtained from SAXS, including surface area (SSA), porosity (ɸ), average chord length of the pores (*l*_pore_), and average chord length of the walls (*l*_solid_). The surface area and porosity of the pure carbon materials align well with the N_2_ physisorption data. Furthermore, *l*_solid_ decreases with increasing activation time, which is expected as prolonged CO_2_ activation erodes more of the carbon surface. Notably, for all hybrid materials, higher IL loadings result in a decrease in surface area, porosity, and *l*_pore_.

For the AC30 hybrid samples, the IL configuration is further supported by the observation that P_4448_ eFAP hybrid samples exhibit higher porosity than EMIM TFSI hybrids, confirming that P_4448_ eFAP covers the pore openings, creating closed porosity. Additionally, as the IL amount increases, the average distance between the walls (*l*_pore_) decreases, while the average thickness of the carbon material (*l*_solid_) increases, indicating a reduction in accessible pore space. In combination with the gas sorption data at ambient or near‐ambient temperatures, this analysis showcases the possibility to tailor pore sizes in materials in a nearly stepless way by functionalizing the pore walls with ILs of appropriate molecular dimensions.

For the AC120 hybrid samples, both SSA and porosity decrease with increasing IL loading. Larger IL molecules and higher IL loadings increasingly occupy the pores, resulting in shorter average distances between the carbon walls while increasing the average distance between the pores, as observed in the AC30 sample. However, the expected trend for porosity based on IL configuration is not fully captured in the SAXS measurements. The detailed data provided by SAXS indicates that this behavior may be influenced by factors beyond pore geometry and IL‐distribution within the sample, involving more complex chemical interactions, like intrinsic gas solubility, polarity, or viscosity of the samples, necessitating a more in‐depth investigation in future work.

**Figure 7:**
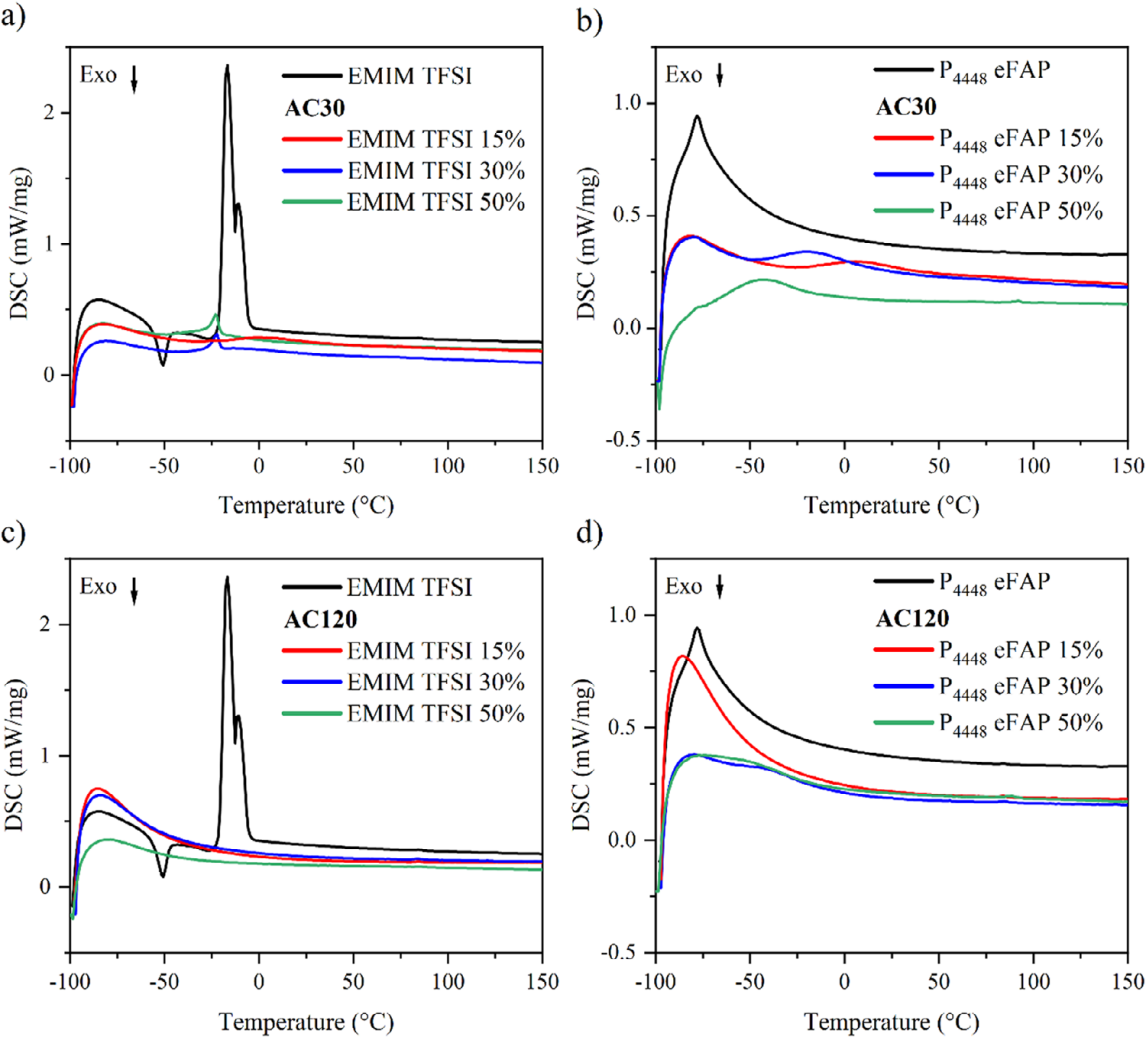
Differential scanning calorimetry (DSC) curves for ionic liquids and for a) AC30 with EMIM TFSI hybrids, b) AC30 with P_4448_ eFAP hybrids, c) AC120 with EMIM TFSI hybrids, and d) AC120 with P_4448_ eFAP hybrids.

Differential scanning calorimetry (DSC) analysis was conducted for the bulk IL and the hybrid materials, as shown in **Figure** **7**, to observe possible phase transitions of the liquids. Structural rearrangements within condensed matter usually require a certain amount of atoms or molecules close to each other, and hence should be affected by pore confinement. The DSC curve for bulk EMIM TFSI reveals a melting point ≈−17 °C and a crystallization temperature near −54 °C, while bulk P_4448_ eFAP displays a melting point ≈−70 °C. Cooling cycles are not shown here, as no distinct peaks were observed during cooling (Figure S9, Supporting Information). Figure 7a,b illustrate the changes in the melting points of the confined liquids compared to those of the bulk IL. This change in melting point can be attributed to the structural rearrangements of the ionic liquid within the carbon, which is known to disrupt the regular ionic interactions observed in the bulk phase.^[^ ^31^ ^]^ Furthermore, interactions between the IL and the carbon surface, such as adsorption, further modify the thermal properties of the IL, leading to the observed shift in transition temperatures. For the AC30‐EMIM TFSI samples (Figure 7a), IL loadings of 30% and above result in the complete filling of the pores, with excess IL covering the external surface and leading to the dominance of bulk IL phase transitions. In contrast, Figure 7c,d show no detectable phase transition for the hybrid materials within this temperature range. As mentioned earlier, the fact that phase transitions and other phenomena such as freezing, recrystallization, or glass transition are collective events between numerous ions qualifies DSC as a unique bulk analysis tool to measure if ions are isolated from each other as a result of pore confinement or not. When considering the IL configuration – where the IL partially fills the pores in the open porosity structure – the absence of detectable phase transitions suggests that there are not enough ions interacting in the vicinity to produce observable peaks, consistent with the expected behavior.

## 3 Conclusion

In this work, a series of CO_2_‐activated microporous carbons with micropore sizes ranging from 0.35 nm to ≈0.8 nm and varying porosities were synthesized to investigate interface interactions when loaded with two different ionic liquids (EMIM TFSI and P_4448_ eFAP) at varying loadings, along with their adsorption properties. From the N_2_ sorption measurements at 77 K, it was observed that EMIM TFSI is more readily accommodated by relatively large micropores, preserving open porosity, whereas no open porosity was observed in other cases. This highlights that matching the pore size with the IL size and loading is crucial for maintaining porosity, which remains accessible for gases at ambient or near‐ambient temperatures.

CO_2_ physisorption at 273 K and N_2_ physisorption at 298 K revealed significant gas uptake. Hybrid materials with P_4448_ eFAP exhibited higher gas uptake than those with EMIM TFSI, indicating distinct IL configurations within different pore structures. EMIM TFSI in AC30 covers the surface of the pores at 15% loading, whereas at higher loadings, capillary forces draw the IL into the pores, transitioning from surface coverage to complete pore filling, leaving no open porosity. In contrast, notable open porosity is maintained in AC120. Conversely, P_4448 _eFAP covered the pore openings, creating closed porosity, which enabled gas molecules to occupy residual pore spaces, resulting in higher gas uptake. These findings demonstrate that the IL configuration within the pores plays a critical role in determining gas uptake behavior.

EMIM TFSI in smaller pores exhibited a pronounced confinement effect, leading to unique thermal properties alongside adsorption behavior. These findings were further supported by complementary analyses, including XRD and DSC, which provided consistent insights into IL configurations and phase transitions. The phase transition behaviors of hybrid samples were significantly altered due to confinement, whereas no phase transitions were observed for AC120‐EMIM TFSI samples due to the lack of sufficient ions in the vicinity of the pore to interact with each other. Additionally, the analyses confirmed that higher IL loadings result in bulk‐like behavior of the IL.

These results provide valuable insights into the modification of pore structures using ionic liquids and their tailored properties at the solid‐liquid‐gas interface. Our study offers new opportunities for designing material combinations with tailored 3‐phase boundaries for electrocatalytic applications such as NRR.

It should be noted that the conclusions drawn about the distribution of ionic liquids in the pores are based on observations from adsorption trends, XRD, and SAXS. While these methods provide consistent and complementary evidence, advanced techniques such as solid‐state NMR or contrast‐matching SAXS/SANS would be required to directly resolve these distributions. Future work will focus on the nitrogen doping into the carbon framework to stabilize surface charge distributions. This strategy is expected to improve the compatibility of the samples with solid‐state NMR technique, potentially enabling direct observation of the arrangement of ionic liquids within the pores.

## 4 Experimental Section

### Materials synthesis—Porous Carbon

5 g of microcrystalline cellulose (Sigma–Aldrich) was carbonized at 900 °C for 1 h under argon flow 5 (Nl h^−1^) in a horizontal tubular furnace with a heating rate of 10 °C min^−1^. The carbonized materials were subsequently activated under CO_2_ flow (5 Nl h^−1^) for 30 min and 120 min, respectively, and subjected to a further heat treatment at 900 °C under argon flow (5 Nl h^−1^) for 30 min before cooling to room temperature.

### Materials synthesis—Ionic Liquid

EMIM TFSI (1‐ethyl‐3‐methylimidazolium‐bis(trifluormethylsulfonyl)imid, ≥98%) was purchased from Sigma–Aldrich.

P_4448 _eFAP (tributyloctyl phosphonium‐tris(pentafluoroethyl) trifluorophosphate) was synthesized by stirring 0.0589 mol of 1‐ethyl‐3‐methylimidazolium tris(pentafluoroethyl)trifluorophosphat (EMIM eFAP, 99%, iolitec) and 0.0366 mol of tributyloctylphosphonium chloride (P_4448 _Cl, >95% purity, iolitec) in a 1:1 (v:v) mixture of dichloromethane (CH_2_Cl_2_):H_2_O for 45 min at room temperature. The organic phase was separated, washed with ultrapure water for five times, and dried in the rotary evaporator. Lastly, the synthesized IL was dried under high‐vacuum conditions overnight.

### Materials synthesis—IL Impregnation

50 mg porous carbon and the calculated amount of ionic liquid (15%, 30%, and 50% of the total pore volume) were mixed in 10 ml ethanol (absolute, ≥ 99.8%, Sigma–Aldrich) and stirred for 30 min in the rotary evaporator and then the solvent evaporated under vacuum and lastly dried in the vacuum oven at 70 °C overnight.

An example was shown for 50 mg AC120 + 15% IL:

(1) $$V_{\mathrm{total}\;\mathrm{pore}}=0.4050\frac{cm^{3}}{g}$$

(2) $$0.05g\times \frac{0.405cm^{3}}{g}=0.2025cm^{3}\left(\mathrm{pore}\;\mathrm{filling}\;100\%\;\right)$$

(3) $$0.2025cm^{3}\times 0.15=0.00304cm^{3}IL\left(\mathrm{pore}\;\mathrm{filling}\;15\%\right)$$

### Characterization of Materials

N_2_ and CO_2_ physisorption measurements were performed on a static volumetric surface area & pore size analyzer (Vapor200C, 3P Instruments). Prior to each measurement, pure carbon samples and IL containing samples were outgassed at 120 °C and 70 °C, respectively, under vacuum for 24 h. To determine the surface area and the pore size distribution of pure carbons, quenched‐solid density functional theory (QSDFT, for N_2_ at 77 K on carbon with slit/cylindrical pores, adsorption branch kernel) was used for N_2_ at 77 K, and non‐local density functional theory model (NLDFT) for the CO_2_ at 273 K to determine the volume and distribution of ultramicropores.

Powder X‐ray diffraction (P‐XRD) patterns of the materials were collected on a Bruker D2 Phaser using Bragg‐Brentano geometry and a horizontal silicon single‐crystal holder, within a 2θ range of 10 to 80 ° and a step size of 0.6 °, utilizing CuK_α_ radiation.

^1^H, ^19^F, and ^31^P NMR measurements of P_4448_ eFAP ionic liquid was performed on Bruker Avance NEO 500. ^1^H NMR (500 MHz, DMSO *d*_6_) 𝛿_ppm_: 0.83–0.86 (3H, t), 0.89–0.92 (9H, t), 1.24–1.29 (8H, m), 1.37–1.47 (16H, m), 2.11–2.17 (8H, m). ^31^P NMR (202 MHz, CDCl) 𝛿_ppm_: 33.43 (1P, s), −156.26–(−139.52) (1P, (q)m). ^19^F NMR (470 MHz, CDCl) 𝛿_ppm_: −45.34–(−43.41) (1F, (d)m), −79.77(3F, m), −81.56(6F, m), −86.52(F, m), −88.61(F, m), −115.55–(−116.25) (6F, (d)m).

Infrared spectra of P_4448_ eFAP ionic liquid (Figure S1, Supporting Information) were measured with a Shimadzu IR‐Spirit spectrometer using a diamond ATR attachment, in the range of 500 to 3500 cm^−1^, accumulating 32 scans with a resolution of 4 cm^−1^. ATR‐IR and ^1^H‐NMR spectrums can be found in the supporting information. (Figure S1, Supporting Information).

Small‐angle x‐ray scattering (SAXS) measurements were performed with a SAXSpoint 5.0 (Anton Paar, Graz, Austria) equipped with a Primux 100 microfocus X‐ray source (Cu Kα radiation; λ = 1.54 Å), ASTIX 2D multilayer X‐ray optics, and a 2D EIGER2 R 1 M hybrid photon counting detector shielded with a mylar film (Dectris, Baden, Switzerland). The SAXS data were processed by applying corrections for sample transmission, background signals, and the sensitivity of the scattering detector. Following normalization to the incident photon flux, the scattering curves were calibrated and expressed in terms of macroscopic scattering cross‐section units, measured in cm^−1^.

Differential scanning calorimetry measurements were conducted using a DSC 204 F1 Phoenix (Netzsch, Selb, Germany). A sample of 10 (±3) mg in an aluminium pan with a pierced lid was measured at a heating/cooling rate of 10 K min^−1^ over a temperature range of −110 to 200 °C at 5000 µV. The data were analyzed using Proteus software.

For the investigation of samples, a high‐resolution field‐emission Scanning Electron Microscope (FE‐SEM) JEOL JSM‐6700F (JEOL Ltd., Tokyo, Japan) was used. In most cases the energy of the exciting electrons, E_0_, was set to 3 keV to ensure a sufficient surface sensitivity. Micrographs were taken with both, Everhardt‐Thornley and in‐lens type detectors. Beyond that, X‐Ray mappings were performed in order to visualize the element distribution. An energy dispersive silicon‐drift detector (EDX; SDD XFlash7100, BRUKER Nano GmbH, Berlin, Germany) and the Esprit evaluation software package were used for this purpose. For a sufficient excitation of the characteristic radiation of the elements in question, the electron energy was slightly increased to *E*_0_ = 5 keV. The sample preparation was carried out by simply sprinkling the powder onto a sticky carbon pad and blowing off excess material. Because of sufficiently good material conductivity, no further coating was necessary.

## Conflict of Interest

The authors declare no conflict of interest.

## Supporting information



Supporting Information

## Data Availability

The data that support the findings of this study are openly available at https://doi.org/10.22000/9u796r4u38ze7bvu, reference number ITUC‐1.
