# Supplementary material for: Understanding the Interplay Between Pore Structure and Ionic Liquid Interaction on the Gas Uptake of Microporous Carbons
Source: Small. 2025 Aug 11;21(37):e01928. doi: 10.1002/smll.202501928 (PMC12444832; doi:10.1002/smll.202501928)
Supplement: Supplementary file 1 — Supporting Information [file SMLL-21-e01928-s001.docx]

Supporting Information

**Understanding the interplay between pore structure and ionic liquid loading on the gas uptake of microporous carbons**

*Merve Ayyildiz, Kai Hetze, Konstantin Schutjajew, Purushottam Poudel, Renzo M. Paulus, Felix H. Schacher, Jan Dellith, Ulrich S. Schubert, Martin Oschatz**


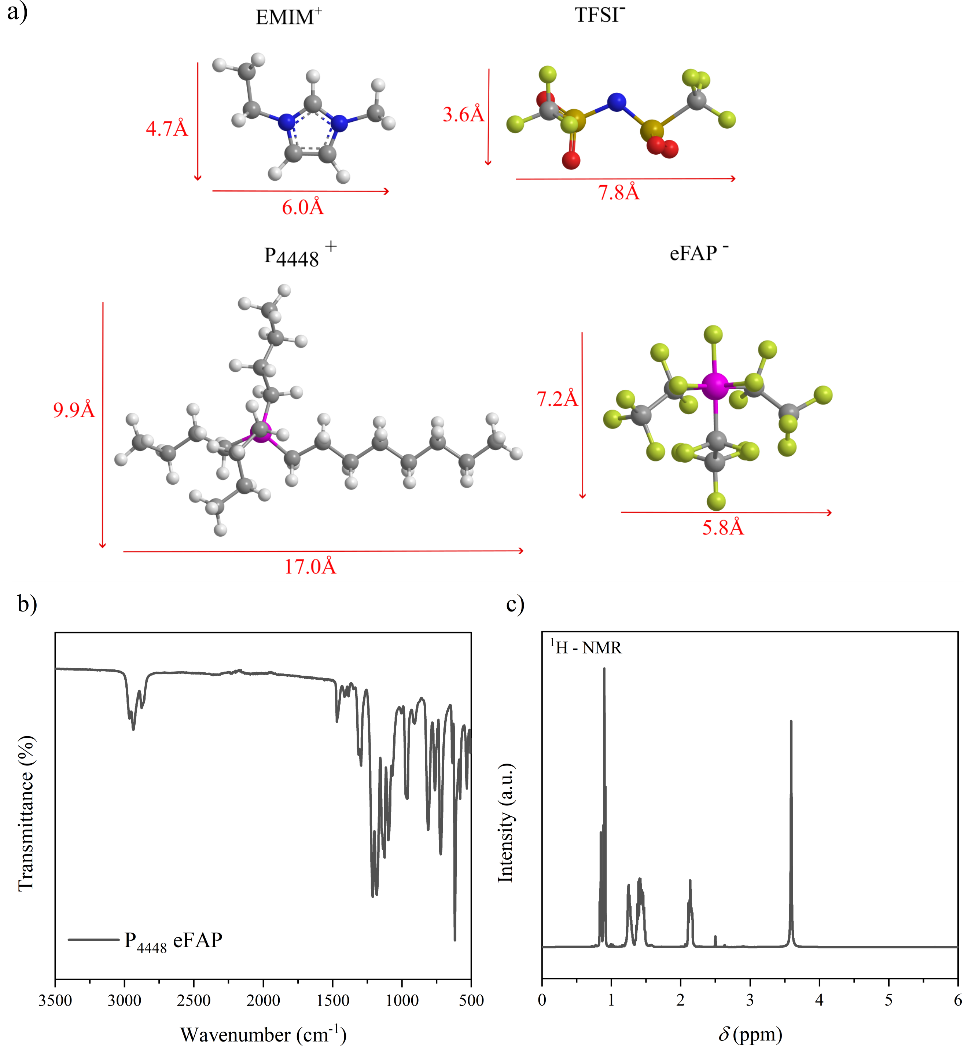


**Figure S1.** a) Structures and dimensions of the used ionic liquid ions. Carbon atoms are shown in gray, nitrogen in blue, hydrogen in white, sulfur in orange, oxygen in red, fluorine in yellow, and phosphorus in pink, b) ATR-IR spectrum and c) ^1^H-NMR spectrum of P_4448_ eFAP.


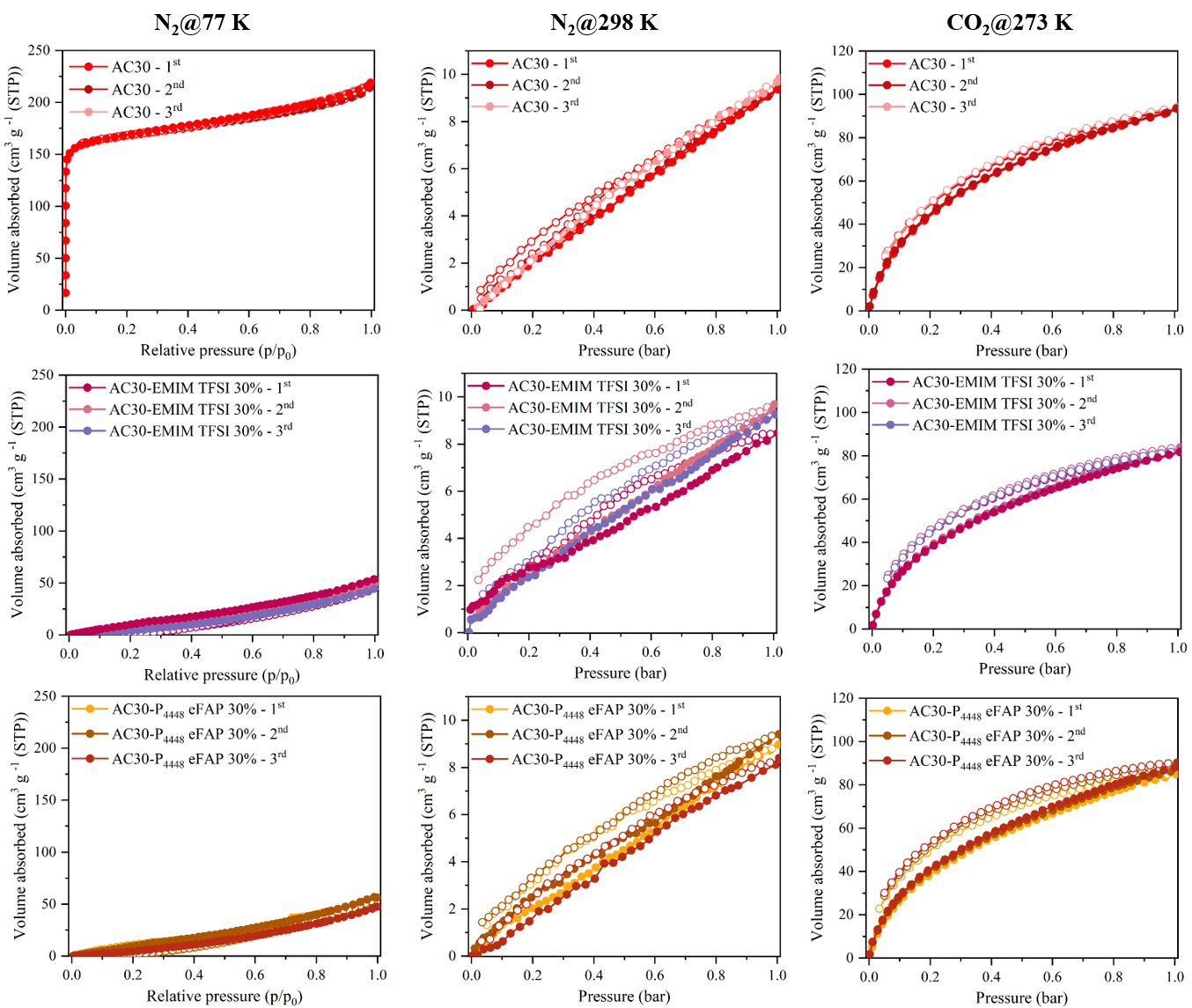


**Figure S2**. Repeat gas adsorption measurements for 3 selected samples; AC30, AC30-EMIM TFSI 30%, and AC30-P_4448_ eFAP 30%, adsorption and desorption branches are represented by filled (●) and open (○) circles, respectively.


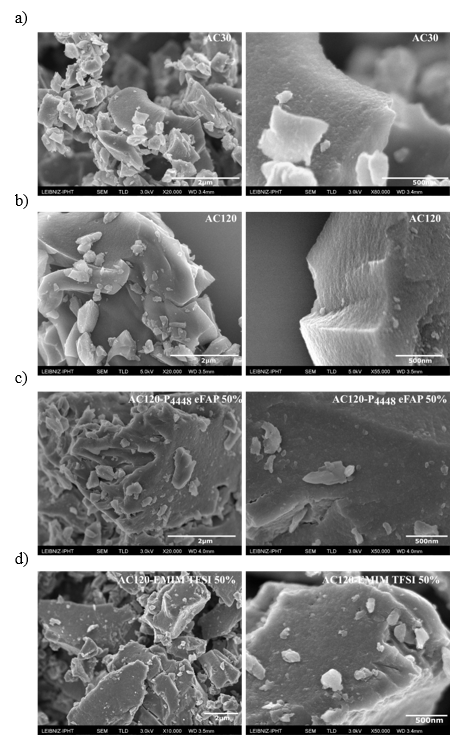


**Figure S3.** Representative SEM images of a) AC30, b) AC120, and AC120 samples loaded with c) P_4448_ eFAP 50%, and d) EMIM TFSI 50%.


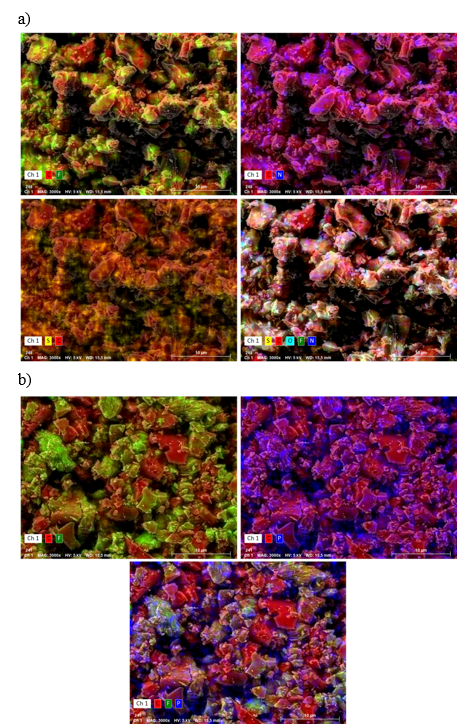


**Figure S4.** EDX mapping images of AC120 samples loaded with a) EMIM TFSI 50% (C-KL_3_ (α) radiation (red), F-KL_3_ (α) radiation (green), N-KL_3_ (α) radiation (blue), O-KL_3_ (α) radiation (light blue), S-KL_3_ (α) radiation (yellow)), and b) P_4448_ eFAP 50% (C-KL3 (α) radiation (red), F-KL3 (α) radiation (green), P-KL_3_ (α) radiation (blue)). (The X-ray signals are superimposed with a secondary electron image (indicator Ch1) to enhance the topographical impression.)

**
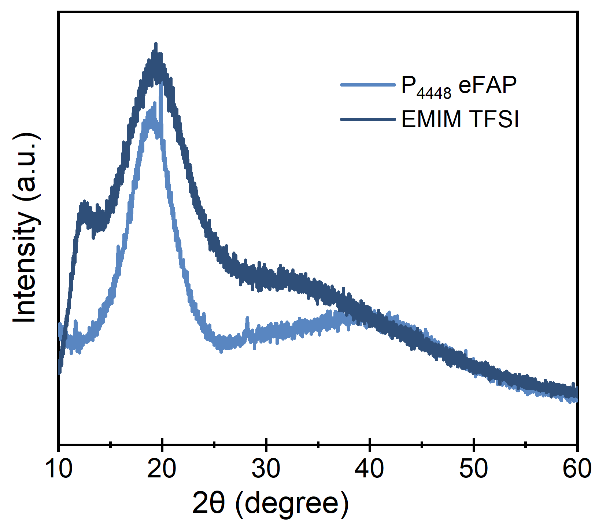
**

**Figure S5.** X-ray diffraction patterns of ionic liquids

**SAXS-Data Reduction**

In the following, we describe the SAXS data analysis method developed by Roland and colleagues ^[1-5]^, which has been recently applied to microporous carbon materials ^[6-8]^.

***Normalization to mass***:

As the material being analyzed is partially in a powdered form and does not completely fill the scattering volume, the absolute scattering intensity is normalized by the apparent filling density ($\rho_{\mathrm{app}}$ ) of the ground powder (see Table S1), giving units of g.cm^-3^.

|  | $\frac{d\Sigma_{m}}{d\Omega}\left( q \right)=\frac{1}{\rho_{app}}\frac{d\sigma}{d\Omega}\left( q \right)$ | (1) |
| --- | --- | --- |

where $q= \left( {4\pi}/\lambda\right)\sin\theta$ is the magnitude of scattering vector with the wavelength λ and the scattering angle $2\theta$.

***Modified Porod fit***:

The macroscopic scattering cross section $\frac{d\sigma}{d\Omega}\left( q \right),$ represents a combination of two distinct scattering contributions. The first, $\frac{{d\Sigma}_{pores}}{d\Omega}\left( q \right)$ , corresponds to the scattering from a two-phase system, such as pores embedded within a carbon matrix. The second term, $\frac{{d\Sigma}_{fluct}}{d\Omega}\left( q \right)$, represents the fluctuation scattering contribution, which is attributed to lateral imperfections within the carbon layers. Additionally, a constant background term, 𝐶 arises from q-independent three-dimensional density fluctuations characteristic of an amorphous phase.

|  | $\frac{d\Sigma_{m}}{d\Omega}\left( q \right)=\frac{{d\Sigma}_{pores}}{d\Omega}\left( q \right)+\frac{{d\Sigma}_{fluct}}{d\Omega}\left( q \right)+C$ | (2) |
| --- | --- | --- |

The lateral imperfection and finite size of the carbon layers is determined by:

|  | $\frac{d\Sigma_{\mathrm{fluct}}}{d\Omega}\left( q \right)= \frac{B_{\mathrm{fl}}{l_{R}}^{2}\left( 18+{l_{R}}^{2}q^{2} \right)}{\left( 9+{l_{R}}^{2}q^{2} \right)^{2}}$, | (3) |
| --- | --- | --- |

The parameter $B_{\mathrm{fl}}$ quantifies the extent of the fluctuation contribution, which predominates at higher q-values. The length $l_{R}$ defines the threshold above which lateral correlations within the carbon layers diminish, potentially due to factors like finite layer dimensions or the bending of the layers. To calculate the fluctuation contribution at high q-values, a modified form of Porod's law is used, which is derived by combining equations (2) and (3).

|  | $\frac{d\Sigma_{m}}{d\Omega}\left( q \right)\overset{\to}{qL\gg1}\frac{{(2\pi)}^{4}P_{m}}{q^{4}}+\frac{B_{\mathrm{fl}}}{q^{2}}+C$, | (4) |
| --- | --- | --- |

Here, $P_{m}$ represent the Porod constant and L corresponds to the structural length scale. When the two-dimensional fluctuation term,$\frac{B_{fl}}{q^{2}}$, becomes the dominant factor, Equation (4) appears as a straight line in the modified Porod plot. However, if a non-zero contribution from 𝐶 exists, the modified Porod plot will display a parabolic shape. The Porod constant is further utilized to calculate the inner surface area, ${SSA}_{\mathrm{SAXS}}$:

|  | $P_{m}= \frac{P}{\rho_{f}}= \frac{{\Delta\rho}^{2}}{{(2\pi)}^{3}}{SSA}_{\mathrm{SAXS}}$ | (5) |
| --- | --- | --- |

Where $\Delta\rho={(N_{A} \rho_{Sk} r_{e} Z)}/{M_{C}}$ is the scattering contrast of carbon vs. vacuum calculated from the skeletal density and where $N_{A}$ is Avogadro’s number, $r_{e}$ the classical electron radius, Z the number of electrons per carbon atom and $M_{C}$ is the atomic weight of carbon. After subtraction of $\frac{{d\Sigma}_{fluct}}{d\Omega}\left( q \right)$eq. (3) and a constant, all corrected scattering curves show the (asymptotic) q^-4^-behaviour as demonstrated in Figure S7.

***Calculation of integral parameters of a two-phase system:***

From the pore scattering $\frac{{d\Sigma}_{pores}}{d\Omega}\left( q \right)$, the invariant is calculated by using following relation

| $Q_{m}= \frac{1}{\left( 2\pi\right)^{3}}\int_{0}^{\infty} \frac{{\text{d}\Sigma}_{pores}}{\text{d}\Omega}\left( q \right)4\pi q^{2}\text{d}q$ | (6) |
| --- | --- |

The invariant $Q_{m}$ is linked to the porosity $\phi$ by:

| $Q_{m}= \frac{\Delta\rho^{2}}{\rho_{grain}}\phi\left( 1-\phi\right),$ | (7) |
| --- | --- |

The term, $\rho_{grain}$refers to the overall mass density of the two-phase system, which includes the carbon matrix and the pores. The grain density $\rho_{grain}$ is higher than the apparent filling density $\rho_{app}$ as the latter also incorporates the empty space between the grains. The relationship between the grain density and the skeletal density $\rho_{Sk}$ is expressed as $\rho_{grain}=\rho_{Sk} (1-\phi)$. The skeletal density of carbon is approximated by 2.0 g.cm^-3^ . ^[6-7]^

|  | $\phi= \frac{Q_{m}}{\rho_{Sk}}\frac{{A_{C}}^{2}}{{N_{A}}^{2} {r_{e}}^{2} Z^{2}}$ | (8) |
| --- | --- | --- |

The average chord length is calculated directly from the scattering results as follows:

|  | $l_{P}=\frac{Q_{m}}{2\pi^{3}P_{m}}=(1-\phi)\left\langle l_{pore} \right\rangle=\phi\left\langle l_{solid} \right\rangle$, | (9) |
| --- | --- | --- |

Where, $Q_{m}$ is the invariant of the system and $\phi$ the porosity. The mean chord length of the pore and solid $\left\langle l_{pore} \right\rangle$ and $\left\langle l_{solid} \right\rangle$ are calculated as follows, respectively:

|  | $\left\langle l_{pore} \right\rangle=\frac{l_{p}}{1-\phi}$; $\left\langle l_{solid} \right\rangle=\frac{l_{p}}{\phi}$ | (10) |
| --- | --- | --- |

**
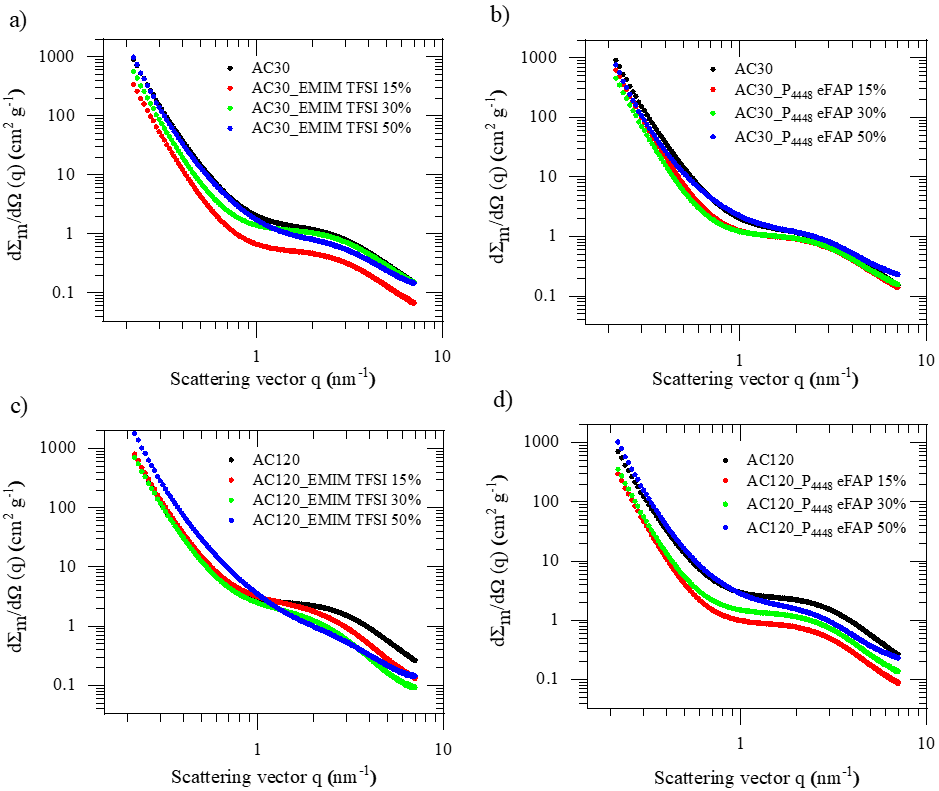
**

**Figure S6**. Normalized scattering curves according to Equation 1: (a)-(b) for AC30 samples, and (c)-(d) for AC120 samples.


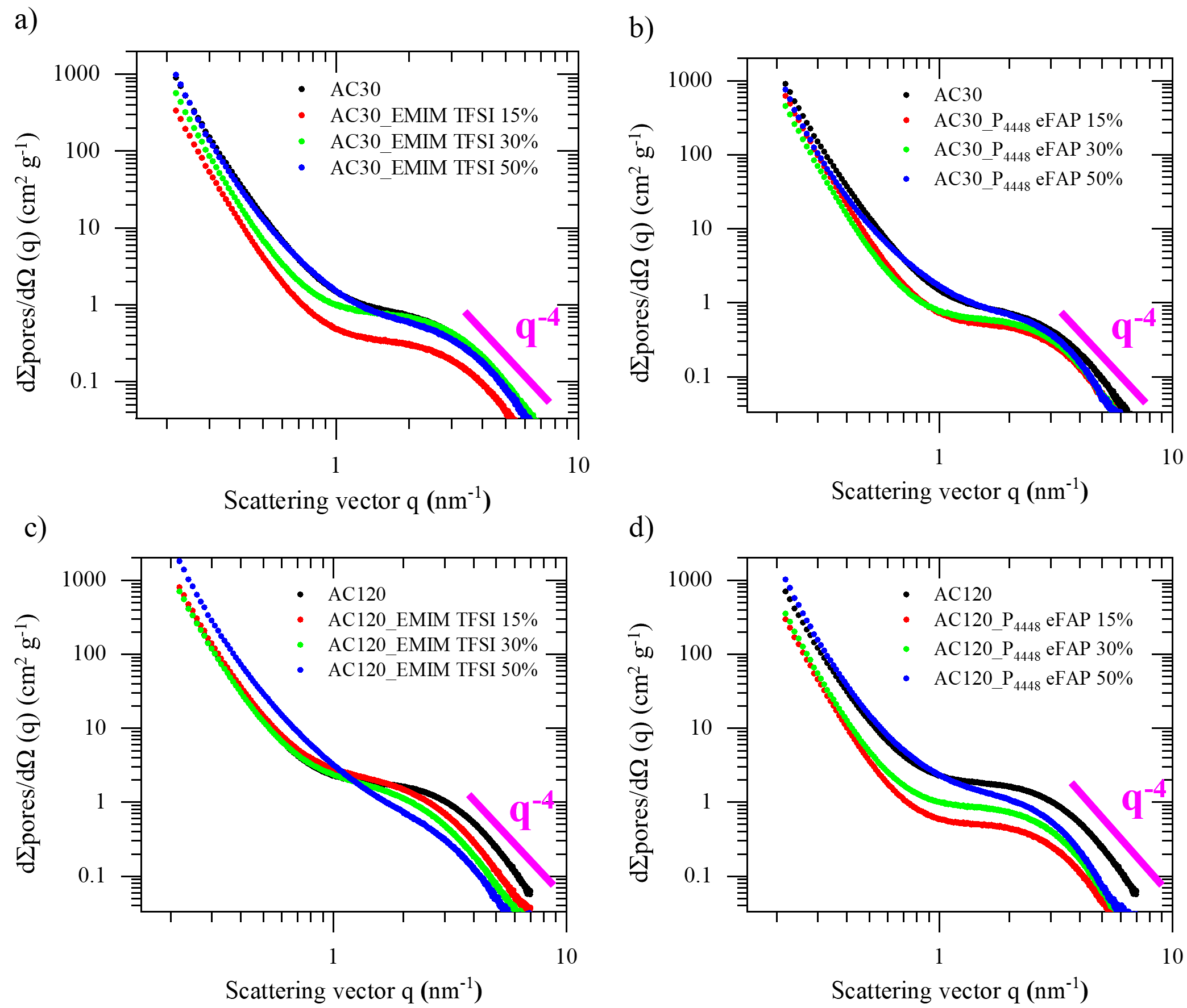


**Figure S7.** Corrected scattering curves: (a)-(b) for AC30 samples, and (c)-(d) for AC120 samples. The fluctuation-induced contribution has been subtracted from the measured normalized scattering intensities. Therefore, the scattering patterns shown here are solely related to the pores in the two-phase system. For all samples (both AC30 and AC120), the final slope of q⁻⁴ is recovered at high scattering angles (see the discussion of Equation 4 above).


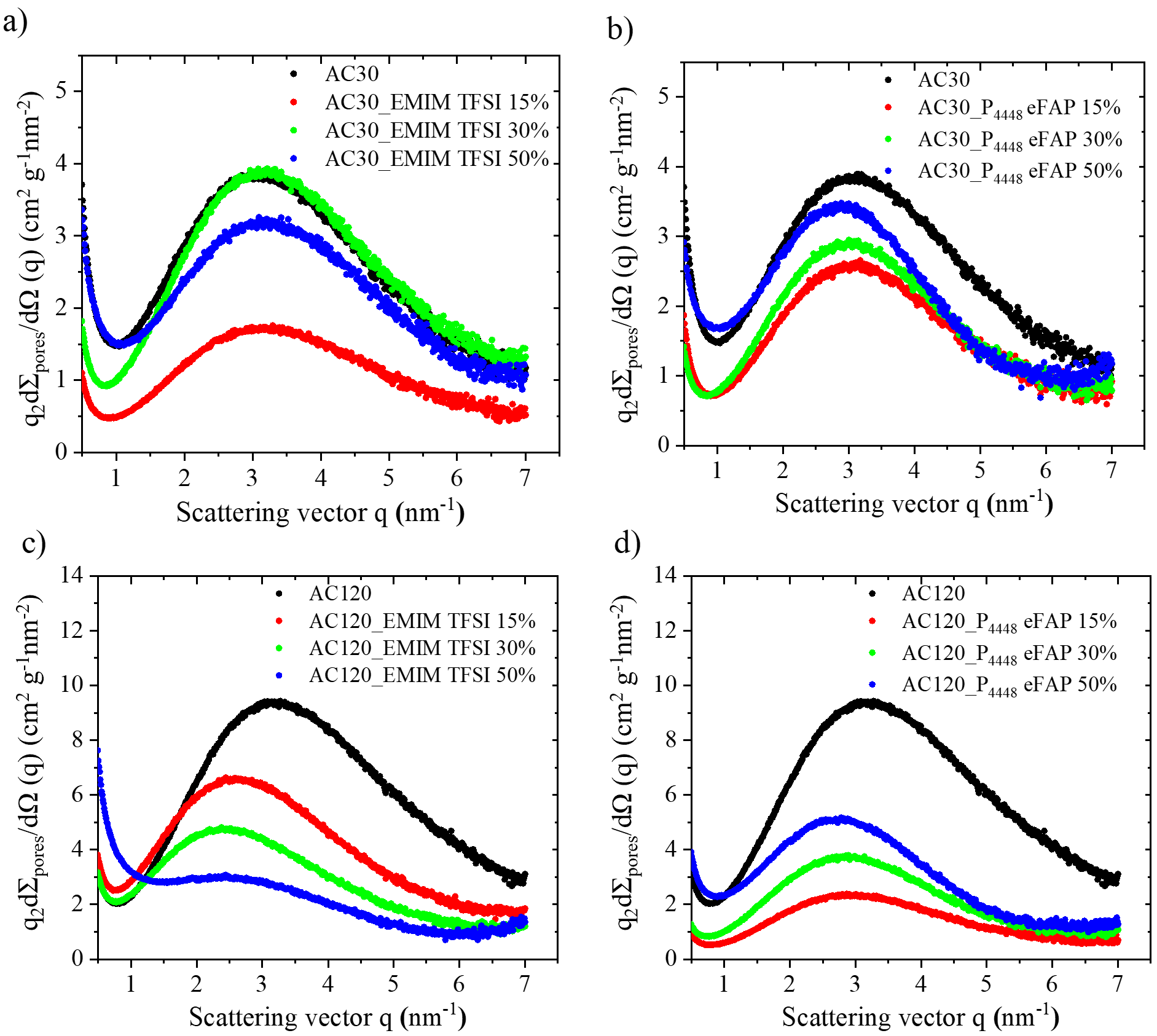


**Figure S8.** Kratky plot of the pore scattering data from Figure S7. (a)-(b) for AC30 samples, and (c)-(d) for AC120 samples.

**Table S1.** Evaluated SAXS characteristics: The scattering contribution B_fl_ (eq.4), Porod constant P_m_ (eq.5), inner surface area SSA_SAXS_, invariant Q_m_ (eq.6), porosity ϕ (eq.8), lateral correlation length l_R_, number-average chord length l_P_ (eq.9), mean chord length of the pore $\left\langle l_{pore} \right\rangle$ and the carbon matrix $\left\langle l_{solid} \right\rangle$ (eq.10) for carbon IL interfaces.

|  | AC30 | AC30_EMIM TFSI 15% | AC30_EMIM TFSI 30% | AC30_EMIM TFSI 50% | AC30_P_4448_ eFAP 15% | | AC30_P_4448_ eFAP 30% | AC30_P_4448_ eFAP 50% |
| --- | --- | --- | --- | --- | --- | --- | --- | --- |
| $\boldsymbol{B}_{\boldsymbol{fl}}$*/*  cm^2^g^-1^nm^-2^ | 5.38  ±0.02 | 3.64  ±0.03 | 2.93  ±0.05 | 2.60  ±0.08 | 4.51  ±0.03 | 5.86  ±0.03 | | 7.56  ±0.04 |
| $\boldsymbol{P}_{\boldsymbol{m}}$/  cm^2^g^-1^nm^-4^ | 0.071  ±0.002 | 0.059  ±0.003 | 0.051  ±0.002 | 0.044  ±0.03 | 0.055  ±0.02 | 0.048  ±0.05 | | 0.043  ±0.01 |
| *SSA*_SAXS_/ m²g^-1^ | 608  ±85 | 502  ±88 | 434  ±65 | 375  ±80 | 468  ±60 | 415  ±50 | | 365  ±40 |
| *Q*_m_ /  cm^2^g^-1^nm^-3^ | 4.692  ±0.052 | 3.664  ±0.123 | 2.951  ±0.086 | 2.185  ±0.124 | 3.954  ±0.076 | 3.091  ±0.66 | | 2.652  ±0.071 |
| *ɸ* | 0.33  ±0.01 | 0.26  ±0.04 | 0.21  ±0.03 | 0.15  ±0.05 | 0.28  ±0.03 | 0.22  ±0.01 | | 0.19  ±0.02 |
| $\boldsymbol{l}_{\boldsymbol{R}}$/nm | 1.4  ±0.1 | 1.5  ±0.2 | 1.8  ±0.1 | ˃10 | 1..0  ±0.2 | 0.95  ±0.02 | | 1.2  ±0.3 |
| *l_p_*/nm | 1.06  ±0.01 | 1.0  ±0.2 | 0.93  ±0.03 | 0.80  ±0.03 | 1.16  ±0.11 | 1.02  ±0.02 | | 0.99  ±0.02 |
| *<l*_pore_*>*  */nm* | 1.58  ±0.10 | 1.34  ±0.11 | 1.18  ±0.16 | 0.95  ±0.05 | 1.61  ±0.13 | 1.31  ±0.11 | | 1.22  ±0.15 |
| *<l*_solid_*>*  */nm* | 3.23  ±0.12 | 3.9  ±0.1 | 4.51  ±0.21 | 5.24  ±0.31 | 4.19  ±0.25 | 4.72  ±0.35 | | 5.37  ±0.41 |
| $\boldsymbol{\rho}$_app_ /  g.cm^-3^ | 0.47 | 0.52 | 0.448 | 0.484 | 0.348 | 0.38 | | 0.418 |

|  | AC120 | AC120_EMIM TFSI 15% | AC120_EMIM TFSI 30% | AC120_EMIM TFSI 50% | AC120_P_4448_ eFAP 15% | AC120_P_4448_ eFAP 30% | AC120_P_4448_ eFAP 50% |
| --- | --- | --- | --- | --- | --- | --- | --- |
| $\boldsymbol{B}_{\boldsymbol{fl}}$*/*  cm^2^g^-1^nm^-2^ | 10.04  ±0.03 | 6.26  ±0.03 | 5.81  ±0.04 | 3.35  ±0.10 | 5.27  ±0.01 | 6.56  ±0.02 | 6.9  ±0.05 |
| $\boldsymbol{P}_{\boldsymbol{m}}$/  cm^2^g^-1^nm^-4^ | 0.118  ±0.003 | 0.094  ±0.005 | 0.088  ±0.012 | 0.065  ±0.012 | 0.085  ±0.005 | 0.073  ±0.004 | 0.059  ±0.005 |
| *SSA*_SAXS_/ m²g^-1^ | 1005  ±90 | 801  ±73 | 750  ±100 | 553  ±60 | 724  ±88 | 622  ±90 | 505  ±100 |
| *Q*_m_ /  cm^2^g^-1^nm^-3^ | 6.485  ±0.065 | 4.815  ±0.112 | 3.47  ±0.098 | 3.125  ±0.019 | 3.769  ±0.153 | 2.475  ±0.103 | 2.176  ±0.121 |
| *ɸ* | 0.45  ±0.01 | 0.34  ±0.02 | 0.24  ±0.05 | 0.22  ±0.04 | 0.26  ±0.03 | 0.17  ±0.01 | 0.15  ±0.03 |
| $\boldsymbol{l}_{\boldsymbol{R}}$/nm | 1.25  ±0.03 | ˃10 | ˃10 | ˃10 | 1.25  ±0.11 | 1.30  ±0.03 | 1.35  ±0.02 |
| *l_p_*/nm | 0.89  ±0.03 | 0.83  ±0.02 | 0.64  ±0.02 | 0.78  ±0.04 | 0.72  ±0.04 | 0.55  ±0.03 | 0.60  ±0.02 |
| *<l*_pore_*>*  */nm* | 1.62  ±0.14 | 1.24  ±0.11 | 0.84  ±0.05 | 0.99  ±0.17 | 0.97  ±0.13 | 0.66  ±0.11 | 0.69  ±0.14 |
| *<l*_solid_*>*  */nm* | 1.95  ±0.15 | 2.45  ±0.12 | 2.61  ±0.22 | 3.54  ±0.12 | 2.71  ±0.17 | 3.15  ±0.16 | 3.88  ±0.21 |
| $\boldsymbol{\rho}$_app_ /  g.cm^-3^ | 0.345 | 0.336 | 0.328 | 0.54 | 0.402 | 0.424 | 0.476 |


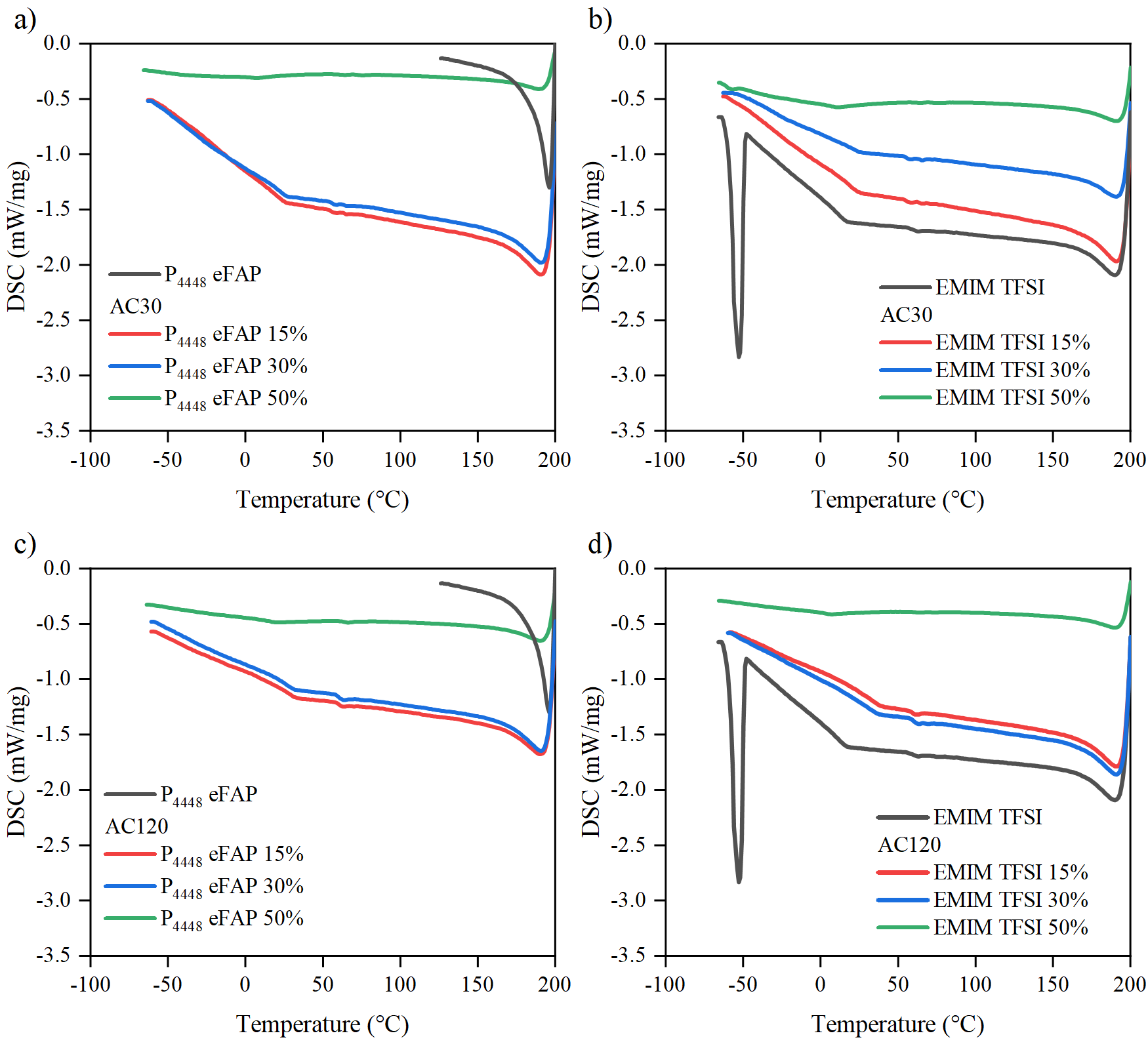


**Figure S9**. Differential scanning calorimetry (DSC) cooling curves for ionic liquids and for a) AC30 with P_4448_ eFAP hybrids, b) AC30 with EMIM TFSI hybrids, c)AC120 with P_4448_ eFAP hybrids, and d) AC120 with EMIM TFSI hybrids.

References

[1] J. Mering and C. Schiller, *Comptes rendus l’Académie des Sci.*, **1967**, 264, 247–250.

[2] R. Perret, W. Ruland, *Journal of Applied Crystallography* **1968**, 1, 308.

[3] R. Perret, W. Ruland, *Journal of Applied Crystallography* **1972**, 5, 183.

[4] W. Ruland, *Advanced Materials*, **1990**, 2, 528.

[5] W. Ruland, *Carbon* **2001**, 39, 323.

[6] C. J. Jafta, A. Petzold, S. Risse, D. Clemens, D. Wallacher, G. Goerigk, M. Ballauff, *Carbon* **2017**, 123, 440.

[7] E. Härk, A. Petzold, G. Goerigk, S. Risse, I. Tallo, R. Härmas, E. Lust, M. Ballauff, *Carbon* **2019**, 146, 284.

[8] E. Härk, A. Petzold, G. Goerigk, M. Ballauff, B. Kent, U. Keiderling, R. Palm, I. Vaas, E. Lust, *Microporous and Mesoporous Materials* **2019**, 275, 139.
